# Preventing clinically relevant pancreatic fistula with combination of linear stapling plus continuous suture of the stump in laparoscopic distal pancreatectomy

**DOI:** 10.1186/s12893-020-00876-8

**Published:** 2020-10-06

**Authors:** Takeshi Aoki, Doaa A. Mansour, Tomotake Koizumi, Kazuhiro Matsuda, Tomokazu Kusano, Yusuke Wada, Tomoki Hakozaki, Kodai Tomioka, Takahito Hirai, Tatsuya Yamazaki, Makoto Watanabe, Koji Otsuka, Ahmed Elewa Abbas Gahin, Masahiko Murakami

**Affiliations:** 1 Division of Gastroenterological and General Surgery, Department of Surgery, School of Medicine, Showa University, 1-5-8 Hatanodai, Shinagawa-ku, Tokyo, 1428666 Japan; 2 General Surgery Department, Cairo University Hospitals, Kasr Alainy, Al-Saray street, El-Manial, Cairo, 11956 Egypt; 3 General Surgery Department, National Hepatology and Tropical Medicine Research Institute, 10. Kasr Alainy street, Cairo, 11562 Egypt

**Keywords:** Pancreatic fistula, Continuous suture for stump closure, Laparoscopic distal pancreatectomy, Stapler closure, Peri-firing compression

## Abstract

**Background:**

Pancreatic fistula is one of the serious complications for patients undergoing distal pancreatectomy, which leads to significant morbidity. The aim of our study is to compare linear stapling closure plus continuous suture with linear stapling closure alone during laparoscopic distal pancreatectomy (LDP) in terms of clinically relevant postoperative pancreatic fistula (POPF) rate.

**Methods:**

Twenty-two patients underwent LDP at our institution between 2011 and 2013. Twelve patients had linear stapling closure with peri-firing compression (LSC) alone compared with ten patients who had linear stapling closure, peri-firing compression plus continuous suture (LSC/CS) for stump closure of remnant pancreas in LDP. Biochemical leak and clinically relevant POPF were compared between both groups.

**Results:**

POPF occurred in 4 of 12 (33.3%) patients with linear stapling closure while no patient developed a clinically relevant POPF in the triple combination of linear stapling, peri-firing compression plus continuous suture group (*p* = 0.043).1 patient (8.3%) in the LSC group and 5 patients (50%) in the LSC/CS group had evidence of a biochemical leak. There were no significant differences in operative time (188.3 vs 187.0 min) and blood loss (135 vs. 240 g) between both groups but there was a significantly of shorter length of hospital stay (11.9 vs. 19.9 days) in LSC/CS group (*p* = 0.037). There was no mortality in either group.

**Conclusions:**

The triple combination of linear stapling, peri-firing compression plus continuous suture in LDP has effectively prevented occurrence of clinically relevant ISGPF POPF.

**Trial registration:**

The study was retrospectively registered September 30, 2019 at Showa University Ethics Committee as IRB protocol numbers 2943.

## Background

Recent advances in laparoscopic techniques and armamentarium have enhanced the feasibility of laparoscopic pancreatic resections and hence its adoption by many surgeons [1–3]. Mortality associated with laparoscopic distal pancreatectomy (LDP) has steadily decreased to reach less than 2% [4–7], yet, its morbidity continues to be significant mostly due to postoperative pancreatic fistula (POPF) [6–8] originating from the exposed and transected pancreatic parenchyma [8]. Previously, the definition of POPF was highly variable resulting in incidence rates ranging between 0 and 61% [9], yet the introduction of the definitions by the International Study Group of Pancreatic Fistula (ISGPF) and their update have made comparison more reliable [10, 11].

POPF is considered the Achilles heel of LDP, which has prompted the development of a multiplicity of pancreatic transection and closure techniques. During the laparoscopic approach, mechanical stapling devices are very commonly used for transection and simultaneous ductal sealing. However, POPF control could not be effectively achieved even with mechanical stapling devices [6]. Therefore, some surgeons attempted to modify their firing technique to overcome this problem. Nakamura et al. reported their experience with prolonged peri-firing compression (PFC) of endo-staplers in reducing POPF after LDP [12]. In 2011, the DISPACT trial, a large muticenter RCT brought evidence that mechanical stapling is not superior to suture closure of the pancreatic remnant in terms of pancreatic fistula and mortality [13]. A more recent systematic review, a meta-analysis and even the Miami guidelines could not put forth a clear recommendation of a definitely superior pancreatic stump closure technique [14–17]. Despite the abundance of reports on various closure techniques, the rate of pancreatic fistula remains high and the controversy prevails [7].

In this preliminary study, we report on our experience with LDP implementing a triple combination of stapler closure after prolonged compression before and after firing buttressed by suture closure and compare it to conventional stapler closure with peri-firing compression.

## Methods

### Patients

The study was approved by the institutional review board of Showa University Ethics Committee (No. 2943). All the participants were informed and written consent was obtained. The methods were carried out in accordance with the approved guidelines. From January 2011 to December 2013, twenty-two LDP procedures were performed at our institution. Twelve patients had linear stapling closure with prolonged peri-firing compression (LSC) alone compared with 10 patients who had linear stapling closure, prolonged peri-firing compression plus continuous suture (LSC/CS) for stump closure of remnant pancreas in LDP.

### Laparoscopic distal pancreatectomy

**Fig. 1:**
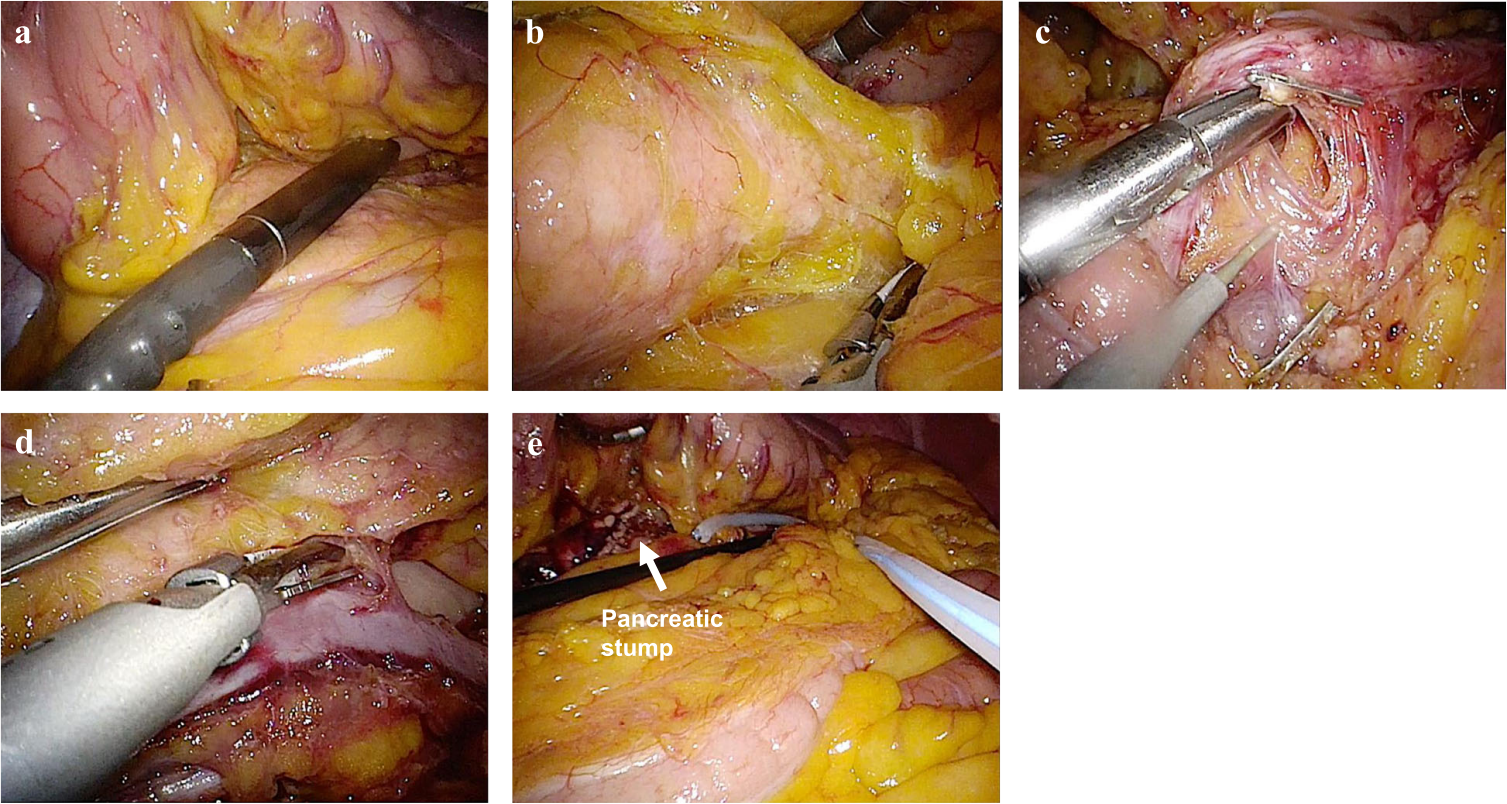
Laparoscopic distal pancreatectomy for intraductal papillary mucinous neoplasm. **a** The tumor was visualized by intraoperative ultrasonography. **b** The pancreas was mobilized from its inferior border off the retroperitoneum. **c** Splenic artery was isolated at the superior pancreatic border, and its branches were dissected off the pancreas and clips are applied to secure them. **d** Splenic vein was isolated from the pancreas, and venous branches were divided and clips were applied for dissection. **e** Drain was routinely placed close to the stump of pancreas

LDP is performed via 5 ports. An umbilical port, a port to the upper right of the mid-line and three ports in the left upper quadrant are inserted. The lesser sac is entered through the gastrocolic omentum and the vasa brevia are secured. The left colonic flexure is mobilized to gain wide access to the lesser sac using a harmonic scalpel. Cephalad retraction of the stomach with its attached greater omentum by a snake retractor adequately exposes the pancreas. Laparoscopic intraoperative ultrasonography is preformed to delineate the exact extent of the tumor (Fig. 1a). We begin pancreas mobilization from its inferior aspect which facilitates dissection of the pancreatic gland off the retroperitoneal structures (Fig. 1b). A retro pancreatic passage is dissected bluntly parallel to the porto-mesenteric axis. The splenic artery is dissected close to its origin along the upper border of the pancreas. The artery is cut and secured with a polymer surgical clip. If a spleen preserving LDP is planned, the splenic vessels are separated from the pancreas (Fig. 1c,d) to allow transection of the pancreatic parenchyma without the vessels. In cases of LDP with splenic preservation but lienal vessel excision as described by Warshaw, the short gastric vessels were preserved. In adherence with oncological safety, spleen preserving LDP was conducted in lesions assumed to be of benign nature. Routine drain placement concluded the intervention (Fig. 1e). Drain output and amylase level were monitored postoperatively. POPF was defined in accordance with the ISGPF. The primary outcome measure was the incidence of clinically relevant POPF (Grade B&C). Mann-Whitney U-test was used in the comparison of patient characteristics and postoperative results between the two groups. A *p* value < 0.05 was considered statistically significant.

### Transection of pancreas

**Fig. 2:**
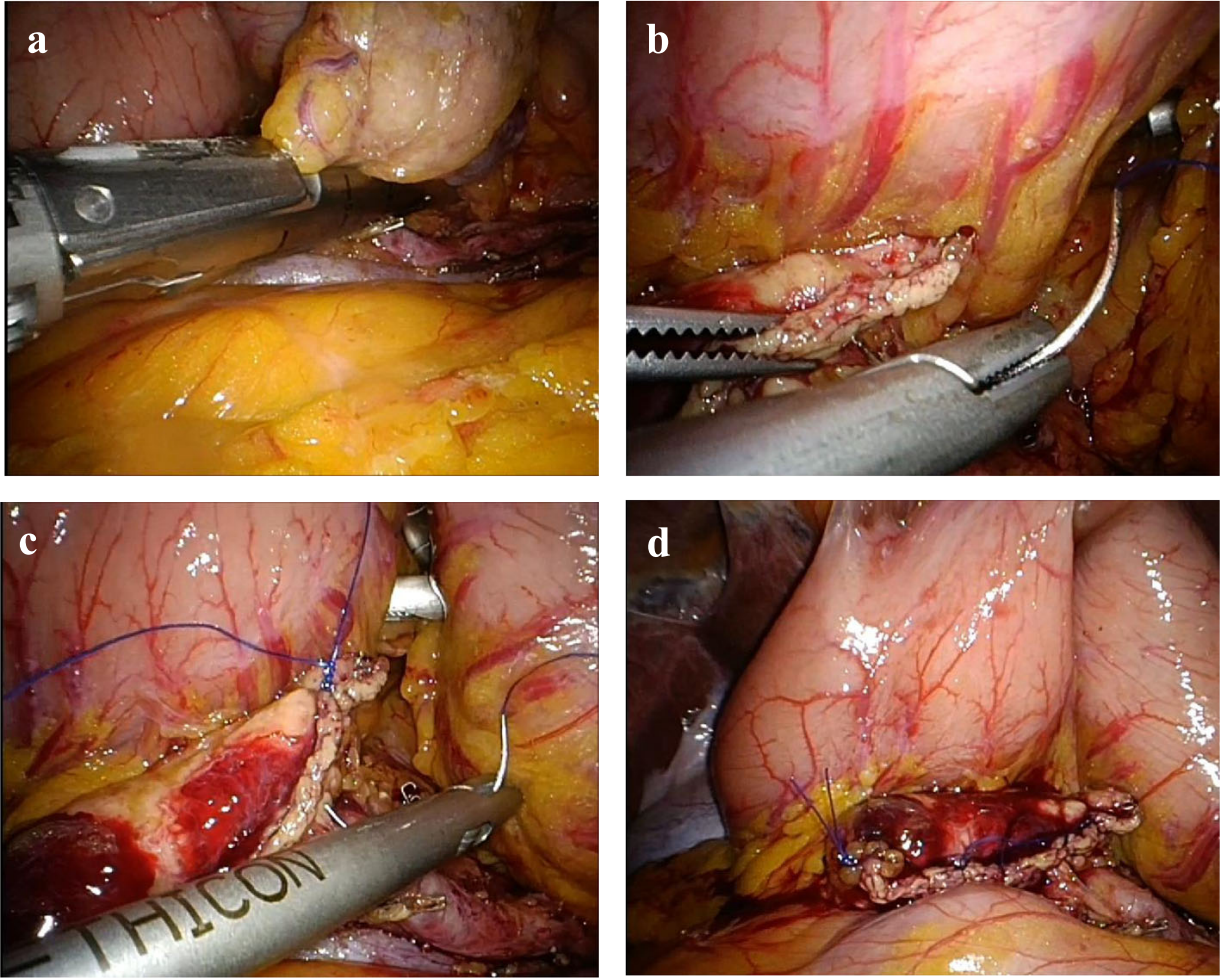
Transection of pancreas and continuous suture for stump closure, **a** After mobilization of pancreas and separation of splenic vessels, the parenchyma of pancreas was transected by linear stapler applying peri-firing compression. **b, c, d** After pancreas transection, continuous suture was added just proximal to the staple line with great care to maintain continuous steady traction throughout the suturing and the regular placement of the suture to achieve equal tension distribution

The neck of the pancreas to the left of the porto-mesenteric axis is gently lifted by a grasper. The division line was dictated by the location of the tumor and a sufficient surgical margin. Transection is performed by an endostapler (Endo Echelon 60 mm stapler with gold cartridge; Johnson & Johnson, Ethicon Endo surgery or EndoGIA; EndoGIA-II 45–4.8 stapler with purple cartridge; Tyco Healthcare, Norwalk, CT, USA) (Fig. 2a). Prolonged peri-firing compression is routinely implemented [12]. Prolonged peri-firing compression consists of 5 min of compression after initial accurate placement and careful gradual stepwise closure of the stapler. Stapler firing is performed very slowly where each 10 mm are fired over 15 s. After completion of the firing the stapler is left in place for 1 further minute before the final cut is initiated and the stapler finally removed. In both LSC and LSC/CS groups the pancreatic parenchyma is transected following these criteria. In LSC/CS a continuous suture (4–0 nylon, Johnson & Johnson, Ethicon Endo surgery) is run just proximal to the staple line (Fig. 2b,c,d) with great care to maintain continuous gentle steady traction throughout the suturing and the regular spacing of the suture to achieve equal tension distribution.

## Results

Twenty-two purely LDP were included in this study. The demographic data of the patients in both groups, the pathology of the pancreatic lesions and pancreatic thickness are demonstrated in Table 1. There were no statistically significant differences between the two groups. There were 2 spleen-preserving LDP in the LSC/CS group and 3 in the LSC group (including 1 case of Warshaw).

**Table 1:** Patient demographics and pathological diagnosis

Variables	LSC/CS (*n* = 10)	LSC (*n* = 12)	*p*-value
Age (years)	67.3 ± 16.5	62.6 ± 16.9	0.574
Gender (male/female)	3/7	2/10	0.457
BMI (kg/m^2^)	22.9 ± 3.2	22.8 ± 2.2	0.947
ASA score(I/II/III)	2/7/1	3/9/0	0.527
Tumor size (mm)	44.7 ± 26.1	40.1 ± 23.3	0.691
Pancreatic thickness (mm)	12.3 ± 3.8	11.8 ± 3.9	0.716
Pathology			
Intraductal papillary mucinous neoplasm	5	1	
Invasive ductal carcinoma	2	3	
Mucinous cystadenoma	1	2	
Neuroendocrine tumor		2	
Serous cystadenoma		1	
Hamartoma		1	
Solid pseudopapillary neoplasm		1	
Others	2	1	

*LSC/CS* Linear stapling closure plus continuous suture, *LSC* Linear stapling closure

Values are presented as mean ± SD

ISGPF Grade B fistula occurred in 4 patients (33.3%) in LSC group and none in LSC/CS group (*p* = 0.043) (Table 2). One patient (8.3%) in the LSC group had evidence of a grade A POPF which according to the update of the ISGPF is now termed biochemical leak [11] while 5 patients (50%) had a biochemical leak in the LSC/CS group. Analysis of the effect of splenic preservation on the POPF rate revealed no statistically significant difference (*p* = 0.150) (Table 3).

**Table. 2:** Surgical outcomes and complications (pancreatic stump closure)

Variables	LSC/CS (n = 10)	LSC (n = 12)	*p*-value
Operative time (min)	187.0 ± 51.4	188.3 ± 55.6	0.947
Blood loss(g)	240.4 ± 220.2	135.0 ± 142.9	0.443
Postoperative hospital stay (days)	11.9 ± 2.9	19.9 ± 11.7	0.037
Postoperative complication(%)≧C-D Grade III^a^ (%)	0 (0)	4 (33.3)	0.043
Pancreatic fistula(%)≧ Grade B^b^ (%)	0 (0)	4 (33.3)	0.043
30-day mortality	0	0	–

*LSC/CS* Linear stapling closure plus continuous suture, *LSC* Linear stapling closure

Values are presented as mean ± SD

^a^Clavien-Dindo Classification

^b^International Study Group of Pancreatic Fistula (ISGPF) classification

**Table 3:** Surgical outcomes and complications (spleen preserving laparoscopic distal pancreatectomy)

Variables	splenectomy (*n* = 17)	spleen-preserving (*n* = 5)	*p*-value
Operative time (min)	180.9 ± 57.0	211.0 ± 23.8	0.306
Blood loss(g)	170.1 ± 181.3	226.0 ± 213.8	0.476
Postoperative hospital stay (days)	14.5 ± 7.1	22.2 ± 15.0	0.157
Postoperative complication(%)≧C-D Grade III^a^ (%)	2 (11.8)	2 (40.0)	0.150
Pancreatic fistula(%)≧ Grade B^b^ (%)	2 (11.8)	2 (40.0)	0.150
30-day mortality	0	0	–

Values are presented as mean ± SD

^a^Clavien-Dindo Classification

^b^International Study Group of Pancreatic Fistula (ISGPF) classification

No mortalities occurred in this study. There were no significant differences in terms of mean operative time (187.0 vs 188.3 min), blood loss (240 vs 135 g), but there was a significantly shorter length of hospital stay (11.9 vs. 19.9 days) in LSC/CS group (*p* = 0.037) (Table 2). No case of stapler closure-related injury occurred in this study.

## Discussion

POPF remains the Achilles heel of distal pancreatectomy in both the laparoscopic and open approach with significant clinical sequelae like intra-abdominal abscess, sepsis or fatal hemorrhage [7]. Various techniques for surgical pancreatic remnant management have been developed to minimize this complication including continuous suturing, staple closure, combinations of staple devices and some kind of stump reinforcement e.g. in the form of sutures or a seromuscular patch, pancreatico- enteric or gastric anastomosis and tissue sealants [7, 14–16, 18]. The multiplicity of suggested techniques is in itself proof of the lack of an ideal solution.

Although pancreatic transection with laparoscopic endostaplers is the most common method, being simple and therefore convenient in LDP, the high rate of POPF is still worrisome. Nakamura et al. compared prolonged PFC in association with the endostapler transection with the conventional staple method in terms of POPF after LDP [12]. Indeed, they report POPF prevention and reduced length of drainage and hospital stay in the PFC group. Seven POPF occurred in the no-PFC group (28%) while no POPF ensued in the PFC group (*p* < 0.05). However, one pseudocyst occurred in each group [12].

This preliminary study was performed to evaluate the effect of combining the endostapler technique with PFC followed by a continuous running suture for stump closure with stapler transection with PFC without suture in POPF reduction after LDP.

In our series, four of twelve patients (33.3%) developed a clinically relevant POPF in the LCS group and 1 patient developed a biochemical leak. On the other hand, five of the ten patients (50%) in the LSC/CS group developed a biochemical leak. Therefore, none of them was clinically relevant (all Grade A) which according to the ISGPF update is no more termed pancreatic fistula. Thus, we postulated that the addition of suturing to stapling with PFC reduces clinically relevant POPF after LDP. This significant improvement in POPF rate after introduction of the additional continuous suture has to be interpreted with some caution as a learning curve effect due to maturation of the technique cannot be clearly excluded. However, the additional continuous suture in LDP was employed in an attempt to lower the high frequency of POPF in the stapler only group.

In the LSC group, two pseudo cysts occurred while in the LSC/CS group no pseudocysts occurred more than 1 month after surgery.

The results of a recent comparative study including 96 distal pancreatectomies performed via either open or laparoscopic access are in harmony with our results. They conclude that LDP is associated with a higher risk of POPF when stump closure is performed with only staplers. On the other hand, adding a continuous suture line to the stapler closure leads to a significant reduction of POPF rate [19]. However, they did not implement PFC.

It is believed that the pathogenesis of POPF after LDP is due to the soft and fragile capsule, parenchyma, and duct of the pancreas. Hence, standard surgical staples rather cut through the parenchyma and multiple small ducts than actually compressing and sealing them [20].

In fact, the only encouraged practice regarding pancreatic stump closure in the Miami guidelines is the gradual compression stepwise closure of the stapler [17] which we achieve through the prolonged peri-firing compression. We believe that the additional careful placement of a continuous running suture with well-maintained gentle steady traction on the suture material creates an equally distributed tight compression on the stump. This equal tension distribution along the closure line seems to be the plausible explanation for the reduced clinically relevant POPF rate observed in our series. Furthermore, there were no significant differences in operative time and blood loss between the two groups in our study.

Several studies and even the Miami guidelines concluded that there was no statistically significant difference in POPF and mortality rates between different pancreatic stump closure techniques [7, 13, 17]. They also did not put forth a clear recommendation in favor of any particular closure technique. Many surgeons consider isolated suture closure of the pancreatic stump technically more demanding in the laparoscopic setting when compared to the open setting especially as very gentle instrumental manipulation of the fragile pancreatic gland is warranted with visual field limitations and spatial orientation issues. Therefore, there is a general trend in favor of the simpler endo-stapler closure in LDP.

We believe that the placement of sutures is significantly simplified after prolonged pancreatic tissue compression and when the staple line is already in place, rendering this triple combination of techniques relatively simple to apply in LDP at the cost of minimal time.

In accordance with the results of this study we adopted the additional continuous suture technique. Our recent (over the last 3 years) grade B postoperative pancreatic fistula rate after LDP is 4.8%. It is worthy of mentioning that very recently, we started to implement it selectively in high-risk cases such as soft pancreas. This shift to selective implementation is due to the significant improvement of linear stapler performance such as electromotion, linear stapling closure with graded and uniform compression and suture reinforcement. Therefore, the majority of the recent LDP procedures at our institution have been performed with powered and buttressed linear stapling technology alone. As the judgement of pancreatic consistency is still very subjective and actually difficult in the minimally invasive approach, we have conducted a study on shear wave elastography to assess pancreatic stiffness and pancreatic exocrine function and their correlation with POPF. This could render judgment as to which cases will benefit most from continuous suturing of the pancreatic stump objective and tailored.

## Conclusions

The primary benefits of our triple stump closure technique seem be the reduction of the incidence of ISGPF Grade B POPF in LDP.

## Data Availability

The datasets generated and analyzed during the current study are available in the figshare repository, https://figshare.com/articles/Continuous_suture_of_the_stump_in_laparoscopic_distal_pancreatectomy_xlsx/12186786

## Abbreviations

LDP: Laparoscopic distal pancreatectomy
POPF: Postoperative pancreatic fistula
ISGPF: International Study Group of Pancreatic Fistula
PFC: Peri-firing compression
LSC: Linear stapling closure with prolonged peri-firing compression
LSC/CS: Linear stapling closure, prolonged peri-firing compression plus continuous suture
